# Double Bladder Sign: Three Cases of an Ultrasonographic Sign that Indicates Ovarian Torsion

**DOI:** 10.7759/cureus.5134

**Published:** 2019-07-13

**Authors:** Judy Lin, Simran Buttar

**Affiliations:** 1 Emergency Medicine, Maimonides Medical Center, Brooklyn, USA

**Keywords:** ultrasound, pocus, ovarian torsion, emergency medicine

## Abstract

Ovarian torsion is a surgical emergency that can be difficult to diagnose and can therefore lead to delayed treatment and loss of ovarian function. While the diagnosis of ovarian torsion is based clinically, several sonographic findings can suggest ovarian torsion, including an enlarged ovary, presence of an ovarian cyst or mass, or abnormal ovarian blood flow. Less commonly described is the finding of an abnormal ovarian location in a clinical setting concerning for torsion. We present three cases of ovarian torsion where an ultrasonographic finding of a “double bladder sign” aided in early detection of ovarian torsion.

## Introduction

Ovarian torsion occurs when the vascular pedicle of the ovary twists and adversely interrupts the blood flow to the ovary, eventually leading to loss of ovarian function, thrombophlebitis and peritonitis [1,2]. Ovarian torsion is the fifth most common surgical emergency and the diagnosis is commonly missed or delayed because the presence of clinical signs and symptoms can be variable and unreliable [3]. The diagnosis is most common in women of reproductive age and therefore reaching a timely diagnosis to avoid loss of ovarian function is critical.

While, several studies have looked at the presence of different sonographic findings in diagnosing ovarian torsion, few have included abnormal ovarian location as a sonographic finding. An abnormal ovarian location is usually described as a midline position of the ovary, usually above the uterus [4,5]. We characterize this abnormal location by describing the “double bladder sign” in three cases, which can help emergency medicine physicians to immediately identify a sonographic finding with a high specificity for ovarian torsion [4].

## Case presentation

Case 1

A 36-year-old woman with past medical history (PMH) of “cysts in her ovaries” presented to the emergency department with the triage complaint of left lower quadrant pain starting an hour and a half prior to arrival. This pain is associated with nausea and vomiting. The nurse noted the patient’s pain to be 10/10 and described it as “terrible pain to the left lower quadrant of the abdomen”. Patient’s last menstrual period was noted as at the end of the September. Her initial vital signs were as follows: blood pressure 159/100 mmHg, pulse 81, respiratory rate 22, and oxygen saturation 98% on room air. Physical exam revealed the patient was in severe pain and distress. Her heart and lung sounds were normal. Her abdominal exam was described as soft, nonperitoneal, severe left lower quadrant tenderness was noted on exam. Genitourinary exam was deferred. The patient received one liter intravenous fluids, and 6 mg of intravenous morphine. The patient still appeared in pain. The attending physician requested a bedside ultrasound from the emergency medicine ultrasound team. The image below shows the “double bladder sign” (Video 1). Obstetrics and Gynecology was consulted. An additional radiology ultrasound was not performed and the patient was taken to the operating room. She was found to have a left adnexal torsion and two left paratubal cysts, the largest measuring 7.5 cm x 7.5 cm x 0.2 cm. Pathology report showed a fallopian tube with hemorrhage and edema. The patient tolerated the procedure well with no complications and was discharged.

**Video 1:** Transabdominal ultrasound demonstrating double bladder sign

Case 2

A 23-year-old female presented with one day history of constant left lower pelvic pain with two episodes of vomiting. Her last menstrual period was three weeks ago. She had a prior history of ovarian cyst that had been followed by her gynecologist and had resolved. She had mild dysuria but no fever, chills, hematuria. Her initial vital signs included a temperature of 97.9°F, pulse of 83, respiratory rate of 18, blood pressure of 112/57, and oxygen saturation of 100% on room air. On exam, the patient appeared in extreme discomfort, and her cardiovascular and pulmonary exams were normal. Her abdomen was soft with tenderness to left lower quadrant but without rebound or guarding. She had localized pain to the left adnexa on bimanual exam without cervical motion tenderness or vaginal bleeding. She received 1 L normal saline and 4 mg of morphine intravenously. Due to her severe pain, a bedside ultrasound was performed showing the following video. The bedside ultrasound showed a “double bladder sign” on pelvic transabdominal sagittal view with the large anechoic ovarian cyst anterior to the uterus and posterior to the bladder (Video 2). Radiology ultrasound showed a left adnexal cyst measuring 6.6 cm x 6.5 cm x 8.1 cm with peripheral follicles and without internal flow. Gynecology was contacted and the patient was taken to the operating room. On laparoscopy there was a left 6-cm paratubal simple cyst, and the left ovary and pedicle was torsed and dusky in appearance. After detorsion of the ovary, the appearance improved and the patient underwent left salpingectomy and removal of the paratubal cyst. The patient tolerated the procedure well and was discharged.

**Video 2:** Transabdominal ultrasound demonstrating double bladder sign

Case 3

A 32-year-old female with no significant PMH presented with one day history of acute left lower quadrant abdominal pain starting at 8 am with 10 episodes of vomiting. Pain was severe at 10/10. She denied fever, dysuria, or hematuria. She reported she had a left ovarian cyst during her previous pregnancy which did not need any intervention. Her initial vital signs included a temperature of 97.4°F, pulse of 62, respiratory rate of 20, blood pressure of 91/54, and oxygen saturation of 108% on room air. The patient had tenderness to left lower quadrant and suprapubic area without rebound or guarding. She received 1 L NS, Toradol 15 mg and morphine sulfate 5 mg IV. Bedside US shows double bladder sign (Video 3). Ob/gyn was consulted and radiology US was performed showing 9.2 cm x 8.2 cm x 5.5 cm left adnexal cyst without vascular flow. The patient was taken to the operating room and through percutaneous endoscopic approach, the left ovary was enlarged, hemorrhagic, dusky, mottled and blue in appearance. After detorsion there was no return of color and she underwent left salpingo-oophorectomy. The patient tolerated the procedure well and was discharged. She was seen on two-week follow-up without complications.

**Video 3:** Transabdominal ultrasound demonstrating double bladder sign

## Discussion

Ovarian torsion is a surgical emergency and its diagnosis is challenging as patients can present with only a few clinical and ultrasonographic findings [1]. Delayed diagnosis can result loss of ovarian function and subsequent oophorectomy or salpingo-oophorectomy. In a menarchal patient, torsion is usually associated with an ovarian cyst which twists along the infundibulopelvic ligament and pedicle, which subsequently compresses the ovarian vein and artery causing stromal edema and ovarian enlargement.

While a majority of studies have found ovarian size to be the most accurate predictor of ovarian torsion, many of these studies did not evaluate ovarian location. Interestingly, Mashiach et al. found that the most specific sign on ultrasound for ovarian torsion was abnormal ovarian location with a specificity of 87.5% [4]. Other less specific findings included: ovarian cyst or mass (Specificity 75%), free fluid around the ovary or pouch of Douglas (Specificity 75%), abnormal ovarian blood flow (Specificity 37.5%), and enlarged ipsilateral ovary (Specificity 18.8%). When an abnormal ovarian location was paired with any one of the other findings, i.e. edema, abnormal blood flow, or enlargement of the ovary, the specificity of ultrasound reached 100%. Therefore, in the setting of high clinical suspicion and other sonographic signs, identification of abnormal ovarian location can greatly improve the specificity of ovarian torsion diagnosis.

We describe the appearance of the abnormal location of the ovary as a “double bladder sign.” The appearance of a midline ovary adjacent to the bladder on a transverse transabdominal scan creates the appearance of two anechoic structures that resembles two bladders visualized in succession. However, the ovarian cyst is also circular in shape and located more superiorly on the image, while the bladder is more rectangular and irregularly shaped and occupies the space inferior to the cyst. It is common for the cyst to be mistaken for the bladder, and for the bladder to be mistaken for free fluid. This occurs because the ovarian cyst is commonly thin-walled and the surrounding ovarian tissue is frequently not clearly seen, especially on a transabdominal image. In addition, there are rarely any follicles within the surrounding tissue to indicate the ovarian origin of the structure. On sagittal view, the ovary lies between the bladder and the uterus and again retains its circular shape, whereas the bladder is more triangularly shaped.

The usefulness of this sign for an emergency physician lies in its potentially high specificity for ovarian torsion as well as the ability to be recognized at the bedside without advanced knowledge of Doppler flow measurements and without needing to perform a transvaginal ultrasound. Characterizing the appearance of an abnormal ovarian location is useful because it may be commonly mistaken for free fluid surrounding the bladder, and should prompt the physician to consider ovarian torsion as a diagnosis. The geographical relationship between the ovary and the ovarian cyst with the bladder may be distorted when visualized on endocavitary ultrasound, and is therefore better seen on transabdominal pelvic ultrasound. Emergency physicians who have a concern for ovarian torsion in a patient should therefore always use a transabdominal approach first to visualize the ovaries before moving to endocavitary ultrasound.

The “double bladder sign” characterizes the appearance of an abnormal ovarian location which may have a high specificity for ovarian torsion. To the best of our knowledge, only Mashiach et al. have looked at the specificity of this sonographic sign and only a few other studies have mentioned this finding [1, 4-8]. Future studies should incorporate an abnormal ovarian location in their description of sonographic findings, and evaluate the specificity of the “double bladder sign” alone and in combination with other clinical and sonographic signs in the diagnosis of ovarian torsion.

## Conclusions

The “double bladder sign” as presented in these three cases can potentially assist the emergency medicine-trained physician in rapid diagnosis of ovarian torsion. In all three of the cases, the diagnosis of torsion is suspected based on history and a point of care transabdominal pelvic ultrasound performed by a credentialed emergency medicine physician. Future studies could explore the specificity and sensitivity of this sonographic sign.
